# A retrospective review of the multidisciplinary management of medullary thyroid cancer: eligibility for systemic therapy

**DOI:** 10.1186/s13044-017-0041-6

**Published:** 2017-09-19

**Authors:** Georgia Geller, Janessa Laskin, Winson Y. Cheung, Cheryl Ho

**Affiliations:** 1 Medical Oncology, British Columbia Cancer Agency, 600 W 10th Avenue, Vancouver, BC V5Z 4E6 Canada; 2 Medical Oncology, Tom Baker Cancer Centre, Calgary, AB Canada

**Keywords:** Medullary thyroid carcinoma, Vandetinib, Cabozantinib, Population-based, Tyrosine kinase inhibitors

## Abstract

**Background:**

Medullary thyroid carcinoma (MTC) accounts for 1-2% of all thyroid cancers. The clinical course of metastatic disease can be indolent. Our aim was to characterize the natural history of disease to evaluate the true proportion of patients who would be eligible for the currently available systemic therapies.

**Methods:**

The British Columbia Cancer Agency (BCCA) provides cancer care to a population of 4.6 million. A retrospective chart review was conducted of all patients with MTC referred to the BCCA from 1991 to 2013. Clinical characteristics, pathology, treatment and outcome data were collected. Relapse free survival and overall survival was determined for patients based on staging at the time of diagnosis.

**Results:**

Of the 98 patients referred to the BCCA during the study period, inherited mutations were found in 6% though 60% did not undergo genetic testing. Based on clinical SEER staging at diagnosis 50% had localized disease, 38% regional, and 12% had distant metastasis. 77% had complete surgical resection of which 25% received adjuvant radiation therapy. Five year relapse free survival (RFS) for localized and regional disease was 75% and 66%, respectively (*p* = 0.006). Initial treatment of 23 patients with locally unresectable and metastatic disease predominantly involved multiple modalities. Of the 37 patients with relapsed or metastatic MTC only 7 (19%) patients received one or more course of chemotherapy for metastatic disease: 1 temsirolimus, 2 adriamycin, 3 sunitinib, 3 sorafenib, and 3 vandetanib. Five year OS based on clinical SEER stage: localized 93%, regional 72% and distant 33% (*p* < 0.001).

**Conclusion:**

Localized and regional MTC treatment patterns reflect multidisciplinary management based on disease characteristics. Patients with distant disease had poor outcomes with 28% of patients dying from disease. In our cohort the minority of patients ultimately received systemic therapy due to timing and lack of TKI availability.

## Background

Medullary thyroid cancer (MTC) is a neuroendocrine malignancy of the parafollicular cells of the thyroid [1]. These cells are responsible for secreting calcitonin, a hormone involved in calcium homeostasis. MTC currently accounts for 2% of all thyroid malignancies [2].

MTC can be associated with an inherited predisposition, with 20-25% of all cases due to mutations in the rearranged during transfection (RET) proto-oncogene. Mutations in RET are associated with autosomal dominant syndromes including MEN2A, MEN2B, familial MTC and are found in approximately 50% of sporadic cases [3].

The cornerstone of local treatment of MTC is surgical resection consisting of total thyroidectomy with dissection of central lymph node compartment and resection of the involved lateral compartment. Radiation has a limited role with little randomized control data supporting its use; however, adjuvant radiation is recommended for microscopic or macroscopic residual disease, extra-thyroidal extension or extensive lymph node metastases and in cases where there is a concern for airway obstruction [4].

Recurrent or metastatic MTC may not be amenable to localized treatment options. The course of disease may also vary significantly with many patients having an indolent course despite metastases. A select group of patients, however, may have more aggressive disease, which may be predicted by the type of RET mutation and the calcitonin doubling time [5, 6].

Up until recently there were no standard first line systemic therapies for inoperable MTC. There is now phase III evidence supporting the use of tyrosine kinase inhibitors (TKIs), vandetanib and cabozantinib, for patients with metastatic disease [7, 8]. Decision-making regarding systemic therapy incorporates not just biochemical parameters but include radiographic findings and the anticipated natural history of the disease.

Our objective was to review the multidisciplinary treatment and outcomes of patients referred to the BC Cancer Agency (BCCA) with MTC to determine the natural history of the disease in our population and the effect of different treatment modalities including surgery, radiation and systemic therapies on patients’ outcomes. With the introduction of systemic therapy into the treatment algorithm, we sought to determine the proportion of patients who would clinically warrant therapy with a TKI.

## Methods

A retrospective review of all patients referred to the BCCA between January 1, 1991 and December 31, 2013 for management of pathologically confirmed MTC was performed.

Patient and tumor characteristics were abstracted from the Outcomes and Surveillance Integrated System (OaSIS) and chart review, including age at diagnosis, gender, RET testing, history of associated MEN2 conditions, presenting symptoms, histology and staging information. The 7th edition TNM system was used for staging. Surveillance Epidemiology and End Results (SEER) program definitions included localized - tumor confined entirely to the thyroid gland, regional -extension beyond the thyroid directly into surrounding tissues or regional lymph node metastases and distant - metastasis to extracervical lymph nodes or organs [9].

Initial and subsequent management including type of surgery, dose, fraction and location of radiation and use of systemic therapy were collected. The disease status at last follow-up, date of death, and cause of death were recorded.

Kaplan-Meier curves for relapse free survival and overall survival were calculated and compared using the log rank test. Relapse free survival was defined as date of complete surgical resection to date of clinical, radiographic or pathologic evidence of recurrence with patients censored for death or date of last follow-up. Overall survival was defined as date of diagnosis to date of death from any cause, with living subjects censored at last follow-up. Cox regression was used to conduct multivariate analysis for OS.

The study was approved by the BC Cancer Agency research ethics board.

## Results

Ninety-eight patients with pathology confirmed MTC were referred to the BCCA during the study period.

Table 1 outlines patient characteristics of the 98 patients. The median age of diagnosis was 54 (range 10-95). The majority of patients were female 53 (54%). Thirty nine patients (40%) had RET testing, with 6 patients (6%) having MEN2 or familial MTC syndrome. Eighty five patients presented with documented localized symptoms which included a neck mass, hoarseness, dysphagia or dyspnea. Twenty patients (20%) had systemic symptoms at presentation including constitutional symptoms, diarrhea, bone pain, or flushing. At baseline 50% of the patients presented with clinically localized disease, 37 patients (38%) with regional disease and 12 patients (12%) presented with metastatic disease based on the SEER classification system.

**Table 1:** Baseline MTC patient characteristics

Patient Characteristics	*n* = 98
Male	45 (46)
Age at diagnosis – median (range)	54 (10-95)
Genetic Mutation – no. (%)	
FMTC or MEN 2	6 (6)
Sporadic	33 (34)
Unknown	59 (60)
Local symptoms at presentation – no. (%)	
Present	85 (87)
Not present	12 (12)
Unknown	1 (1)
Systemic symptoms at presentation – no. (%)	
Present	20 (20)
Not present	77 (79)
Unknown	1 (1)
Clinical Stage at presentation - no. (%)	
Stage I	19 (19)
Stage II	26 (27)
Stage III	11 (11)
Stage IVa	25 (26)
Stage IVb	0 (0)
Stage IVc	12 (12)
Unknown	5 (5)
Clinical SEER at presentation- no. (%)	
Localized	49 (50)
Regional	37 (38)
Distant	12 (12)

**Fig. 1:**
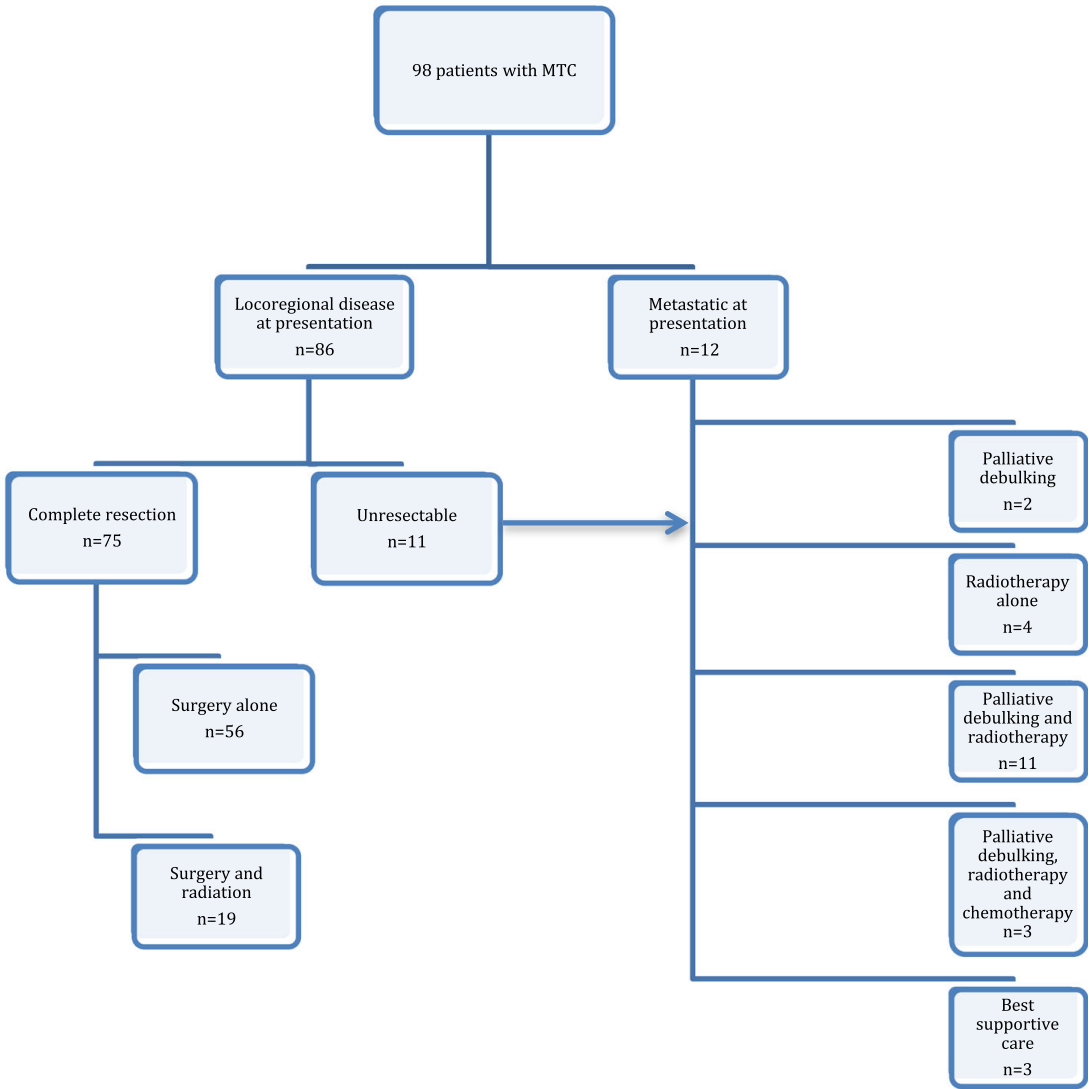
Initial management of MTC

The initial management of patients is outlined in Fig. 1. Of the 86 patients with locoregional disease 75 were resected without evidence of gross residual disease. Surgical intervention included 1 lobectomy, 2 lobectomy with isthmusectomy and 72 total/subtotal thyroidectomy. Nodal management of disease included: 9 no nodal surgery, 31 limited and 35 modified radical/radical neck dissection. Nineteen of these patients also received adjuvant radiation. Sixty nine patients had postoperative calcitonin levels available and of these, 38 patients (55.1%) had normalization of calcitonin and 31 (44.9%) had persistently elevated calcitonin despite resection. The initial treatment of 23 patients with unresectable or metastatic disease were: 2 palliative debulking only, 4 radiation only, 11 palliative debulking and radiation, 3 all 3 modalities and 3 best supportive care alone.

The delivery of systemic treatment is outlined in Table 2. Thirty seven patients either presented with metastatic disease or developed recurrence or metastatic disease. Seven patients received one or more course of systemic therapy and 5 of these patients received one or more TKIs. Of the 27 patients who died from metastatic MTC, 19 died prior to the availability of TKIs.

**Table 2:** Systemic treatment of unresectable or metastatic medullary thyroid carcinoma

Systemic therapy for MTC n = 98	n (%)
Metastatic disease at presentation (%)	12 (12)
Developed unresectable recurrence or metastatic disease (%)	25 (26)
Received systemic therapy for unresectable or metastatic MTC (%)	7/37 (19)
Systemic therapy given^a^ – no.	
Sunitinib	3
Sorafenib	2
Vandetanib	3
Temsirolimus	1
Doxorubicin	2
Death due to MTC – no. (%)	27 (28)
Prescribed TKI	2
Prescribed doxorubicin	2
Discussed TKI but did not receive therapy	5
TKI available but never discussed	1
TKI not available	17

^a^Some patients received 2 or more lines of systemic therapy and are still alive on treatment

**Fig. 2:**
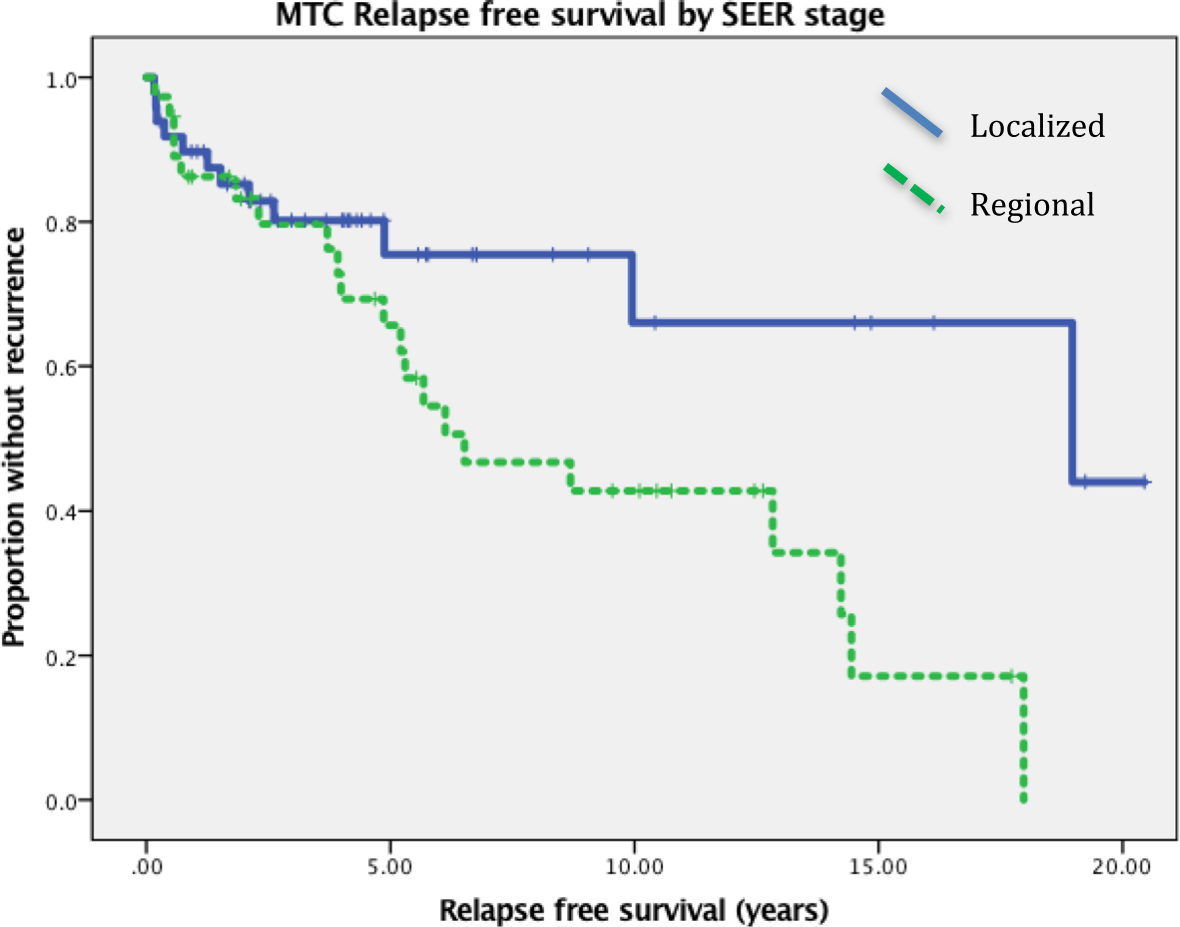
Relapse free survival in MTC patients by SEER stage

**Fig. 3:**
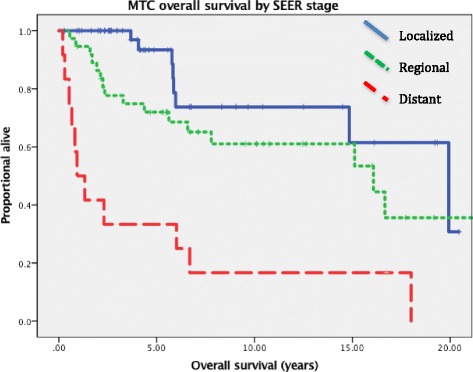
Overall survival for MTC patients by SEER stage

RFS and OS are shown in Figs 2 and 3. Five year RFS for localized and regional disease was 75% and 66%, respectively (*p* = 0.006). Five year OS rates and medians based on clinical SEER stage: localized 93%/19.9 years, regional 72%/16.1 years, and distant 33%/0.9 years (*p* < 0.001). Median OS for all patients at diagnosis was 16.1 years.

Univariate and multivariate analysis showed that older age, systemic symptoms at presentation and more advanced SEER stage were associated with poorer OS (Table 3).

**Table 3:** Univariate and multivariate analysis of the impact of baseline characteristics on overall survival

	Univariate HR	*P* value	Multivariate HR	*P* value
Female	1.58	0.19		
Older age	1.04	<0.001	1.06	<0.001
Local symptoms at presentation	0.54	0.27		
Systemic symptoms at presentation	3.68	<0.001	3.42	0.002
SEER stage				
Regional vs localized	2.19	0.07	3.55	0.007
Distant vs localized	7.76	<0.001	7.15	<0.001

## Discussion

This study confirms the typically indolent nature of MTC with the median OS of all patients being 16.1 years. On multivariate analysis patient age at diagnosis, systemic symptoms at presentation and more advanced stage were associated with poor overall survival. Following the natural history of disease over multiple years demonstrates that 38% of patients would be ultimately eligible for systemic therapy for disease based on the patterns of recurrence and the cause of death.

In our study, similar to other groups, negative prognostic indicators included advanced age, systemic symptoms at presentation and more advanced stage at presentation. The SEER database analysis of over 1200 patients with MTC identified that age > 65 and advanced stage were associated with worse survival outcomes [10]. Two Korean studies also noted that distant metastases portended a reduction in survival [11, 12]. T stage and age were noted in an Irish population to have poorer overall survival [13]. Beyond baseline characteristics, Barbet et al. found that a calcitonin doubling time over 2 years had a 10 y survival over 100%, compared to those with a doubling time less than 0.5 years being only 8% [6]. These variables can help provide some insight into prognosis and may facilitate selection of MTC patients for consideration of therapy.

With the introduction of new systemic therapies for management and the understanding that MTC can often be an indolent disease, our goal was to determine the proportion of patients truly eligible for systemic treatment based on recurrence and death from MTC. The BC Cancer Agency collects all malignant diagnoses in the province as part of our responsibilities for reporting to the Canadian Cancer Registry and provides all of the radiotherapy for a population of 4.6 million. This unique registry organization enables a true population based overview of the natural history of many cancers including MTC.

In our cohort, 38% of patients ultimately would have been eligible for systemic therapy based on disease aspects alone. The true number, however, would likely be lower due to comorbidities and patient preferences based on the side effect profiles of therapy. Due to timing and lack of availability, only 19% of patients had received systemic therapy, these patients were most commonly treated with multi-targeted tyrosine kinase inhibitors (TKIs) focused on vascular endothelial growth factor (VEGF) that were available off-label through a government funded compassionate access program. The positive phase III data for vandetinib and cabozantinib now offer symptomatic patients therapeutic options that improve progression free survival with an acceptable toxicity profile [7, 8].

There are several limitations to this study. It is retrospective in nature and despite serving a large population over a long duration of time there were only 98 patients. Several data points were not available because the data are based on physicians’ usual patterns of practice; they included testing for RET mutations and calcitonin doubling time. While many patients did have serial calcitonin levels it was difficult to calculate a meaningful doubling time due to transition between 3 different laboratory tests over the study period. Our strengths include the population-based nature of the study that allows a longitudinal view of the natural history of disease.

## Conclusions

The natural history of localized and regional MTC is generally indolent with many patients having excellent long-term outcomes. Our population-based study suggests that only a small proportion of MTC patients will require treatment with systemic therapy for palliation over the course of their disease. Baseline factors that may enable appropriate patient selection for systemic treatment are limited and it is the likely the behavior of disease over time with biochemical and radiologic changes being the best means of correctly selecting patients for therapy.
